# Renal Replacement Techniques in Septic Shock

**DOI:** 10.3390/ijms221910238

**Published:** 2021-09-23

**Authors:** Tapio Hellman, Panu Uusalo, Mikko J. Järvisalo

**Affiliations:** 1 Kidney Center, Turku University Hospital and University of Turku, Building 4, AA7, Kiinanmyllynkatu 4-8, FIN-20521 Turku, Finland; tapio.hellman@tyks.fi; 2 Department of Anaesthesiology and Intensive Care, Turku University Hospital and University of Turku, Building 18, TG3B, Hämeentie 11, FIN-20521 Turku, Finland; panu.uusalo@tyks.fi; 3 Perioperative Services, Intensive Care and Pain Medicine, Turku University Hospital and University of Turku, Building 18, TG3B, Hämeentie 11, FIN-20521 Turku, Finland

**Keywords:** sepsis, septic shock, acute kidney injury, renal replacement therapy, cytokine storm, hemoadsorption, high cut-off membrane

## Abstract

Sepsis is defined as a life-threatening organ dysfunction caused by a dysregulated host response to an infection; it carries a risk for mortality, considerably exceeding that of a mere infection. Sepsis is the leading cause for acute kidney injury (AKI) and the requirement for renal replacement therapy (RRT) in intensive care unit (ICU) patients. Almost every second critically ill patient with sepsis will develop AKI. In septic shock, the dysregulated host response to infectious pathogens leads to a cytokine storm with uncontrolled production and release of humoral proinflammatory mediators that evoke cellular toxicity and promote the development of organ dysfunction and increased mortality. In addition to treating AKI, RRT techniques can be employed for extracorporeal adsorption of inflammatory mediators using specifically developed adsorption membranes, hemoperfusion sorbent cartridges or columns; these techniques are intended to decrease the level and early deleterious effects of circulating proinflammatory cytokines and endotoxins during the first hours and days of septic shock treatment, in order to improve patient outcomes. Several methods and devices, such as high cut-off membranes, the Oxiris^®^-AN69 membrane, CytoSorb^®^ and HA380 cytokine hemoadsorption, polymyxin B endotoxin adsorption, and plasmapheresis have been examined in small study series or are under evaluation as ways of improving patient outcomes in septic shock. However, to date, the data on actual outcome benefits have remained controversial, as discussed in this review.

## 1. Definitions and Epidemiology of Sepsis and Septic Shock

Sepsis is a severe multi-organ syndrome caused by an infection and is associated with increased morbidity and mortality. According to the most recent iteration of the Sepsis-3 criteria, sepsis is defined as a life-threatening organ dysfunction associated with a dysregulated host response to an infection [1]. Organ dysfunction in the Sepsis-3 criteria is denoted as a clinical deterioration reflected by an increase of ≥2 points in the Sequential Organ Failure Assessment (SOFA) score in response to infection [2]. Furthermore, septic shock is classified as a subtype of sepsis with more substantial hemodynamic and metabolic deteriorations as well as a more elevated risk of death than in general sepsis [1]. The criteria for septic shock include persistent hypotension necessitating vasopressor medication to maintain a target mean arterial pressure of ≥65 mmHg and a serum lactate level of >2 mmol/l in spite of volume resuscitation. 

According to a recent global survey, sepsis is a common condition worldwide with an age-standardized incidence rate of 677.5 per 100,000 population [3]. Sepsis is associated with a high mortality as 11 million sepsis-related deaths were recorded in 2017, accounting for nearly 20% of all deaths globally [3]. Recent studies have described a hospital mortality rate of 15–30% and a one-year mortality of 35% in patients with sepsis [4,5,6]. Most cases of septic infections originate from the respiratory system and gastrointestinal tract. The causative agents are most commonly Gram-positive bacteria followed by Gram-negative bacteria and fungi [3,7,8], but in some septic patients the causative organism cannot be identified. 

Septic shock, the most severe form of sepsis, is less common and affects 10–20% of sepsis patients admitted to intensive care units (ICUs), corresponding to an estimated incidence of 20 per 100,000 population [4,9]. Septic shock carries a higher mortality risk compared to general sepsis, with observed ICU, hospital, and one-year mortality rates ranging between 37–47%, 39–56%, and 60%, respectively [4,6,9]. 

## 2. Cytokine Storm in Septic Shock

The immune system is responsible for recognizing foreign invaders, responding proportionally to the pathogen burden, and restoring homeostasis back to normal during the infection. Due to constant exposure to external pathogens, the immune system invariably seeks to strike a balance between proinflammatory and anti-inflammatory states. A cytokine storm is a cascade of several adverse immune dysregulation disorders of both pro- and anti-inflammatory cytokines, associated with subsequent collateral organ damage [10]. 

Cytokines are small, usually <40 kDa, proteins released from white blood cells, particularly macrophages, B lymphocytes, T lymphocytes, and mast cells, but also from endothelial cells, fibroblasts, and various stromal cells. Cytokines play a key role as initiators and regulators of stress metabolism [11]; these agents may be categorized into lymphokines (cytokines produced by lymphocytes), monokines (cytokines produced by monocytes), chemokines (cytokines produced by chemotactically active cells), and interleukins (IL; cytokines produced by leukocytes), although there is some overlap in the terminology [12]. The proinflammatory cytokines include, but are not limited to, IL-1 (a family of 11 cytokines), IL-6, IL-17, and tumor necrosis factor α (TNF-α). These compounds activate target cells through specific receptors and stimulate the cells to produce chemokines, proinflammatory cytokines, and other biologically active substances. Subsequently, there are increases in the expression of surface antigens and adhesion molecules in stimulated cells, and therefore enhanced proinflammatory cell–cell interactions [13]. IL-4, IL-10 (family of 12 cytokines), and tumor growth factor β (TGF-β) attenuate the intensity of the inflammatory response and thus strive to prevent any collateral tissue damage caused by excessive cell stimulation and inflammation [14]. The purpose of certain chemokines, such as IL-8, is to attract different types of white blood cells into the targeted peripheral tissue during inflammation to combat the external threat. Once a chemokine binds to the leukocyte surface receptor, the cell becomes activated and undergoes rapid morphologic changes. Then, the chemotactically activated leukocyte penetrates into the tissue between the endothelial cells in the vessel wall and starts to migrate toward the target inflammation process [15]. Interferons fall into three different groups; type I interferons are a family of 20 interferons (e.g., IFN-α, IFN-β, and IFN-ω) which are important for defending against viruses. Type III interferons consist of four different interferons (IFN-λ 1–4) with similar functions to type I interferons [16]. IFN-α inhibits cell growth and division and may thus be used in the treatment of neoplasms [17]. IFN-λ is involved in antiviral defense in the liver and on epithelial surfaces. IFN-γ is a type II interferon, which increases macrophage activation and tissue antigen expression, and can cause prompt symptoms such as chills, fatigue, and fever in a clinical setting [18]. In fact, a monoclonal antibody, emapalumab, which binds IFN-γ, has been approved by the FDA for the treatment of a cytokine storm in patients with primary hemophagocytic lymphohistiocytosis [19].

**Figure 1:**
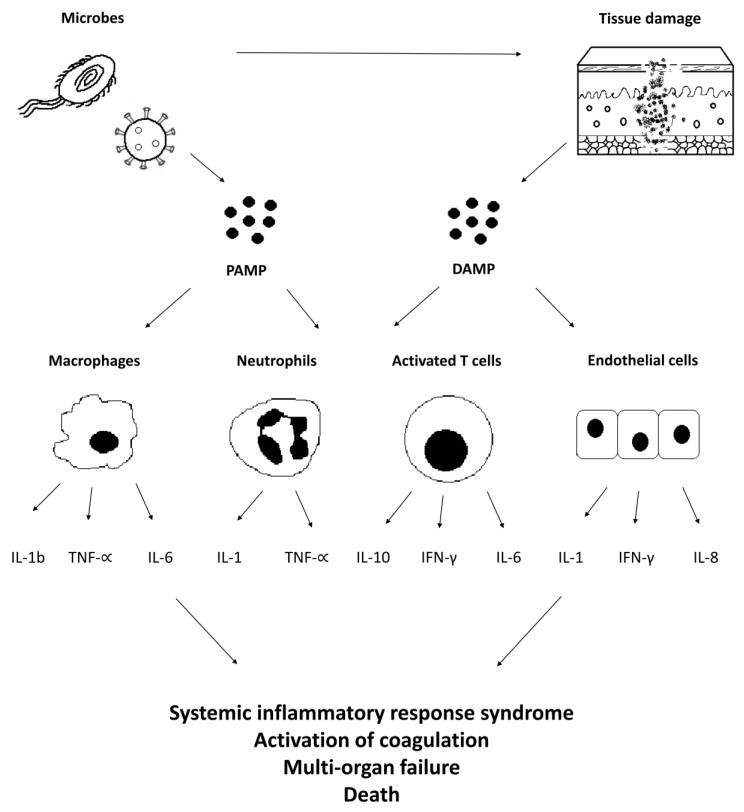
Mechanisms of a septic-shock-induced cytokine storm and associated complications. The presence of pathogenic microbes leads to the detection of pathogen-associated molecular patterns (PAMPs) and damage-associated molecular patterns (DAMPs) that both lead to dysregulated activation of various cells (especially macrophages, T-cells, neutrophils, and endothelial cells). This is followed by the release of proinflammatory and anti-inflammatory cytokines. The dysregulated host response can induce many clinical adverse effects, including systemic inflammatory response syndrome, multi-organ failure, activation of coagulation, or death. PAMP, pathogen-associated molecular pattern; DAMP, damage-associated molecular pattern; IL, interleukin; IFN, interferon; and TNF, tumor necrosis factor.

In septic shock, the detection of pathogen-associated molecular patterns (PAMPs) and damage-associated molecular patterns (DAMPs) leads to the dysregulated activation of a variety of cells (especially macrophages, T-cells, neutrophils, and endothelial cells). This is followed by the release of inflammatory cytokines (Figure 1) as well as adverse tissue and organ damage. The onset and duration of a cytokine storm depend on the causative pathogen and received care [20]. Early symptoms related to a cytokine storm are fever, fatigue, myalgia, arthralgia, headache, rash, diarrhea, anorexia, and neuropsychiatric disorders. Later effects include hypoxemia, dyspnea, hypotension, vasodilatory shock, disseminated intravascular coagulation with vascular thromboses, and/or catastrophic hemorrhages. Severe cases of cytokine storms can lead to renal failure, acute liver injury, stress-related takotsubo-like cardiomyopathy, and/or death [10]. Laboratory findings in cytokine storm include increased levels of CRP, procalcitonin, leukocytosis or leukopenia, thrombocytopenia, anemia, as well as elevated ferritin and D-dimer levels [21,22]. The concentrations of interferon-γ, IL-6, and IL-10 are typically raised during a cytokine storm. However, precise measurement of circulating cytokine levels can be difficult due to the short half-lives of these molecules [10].

**Figure 2:**
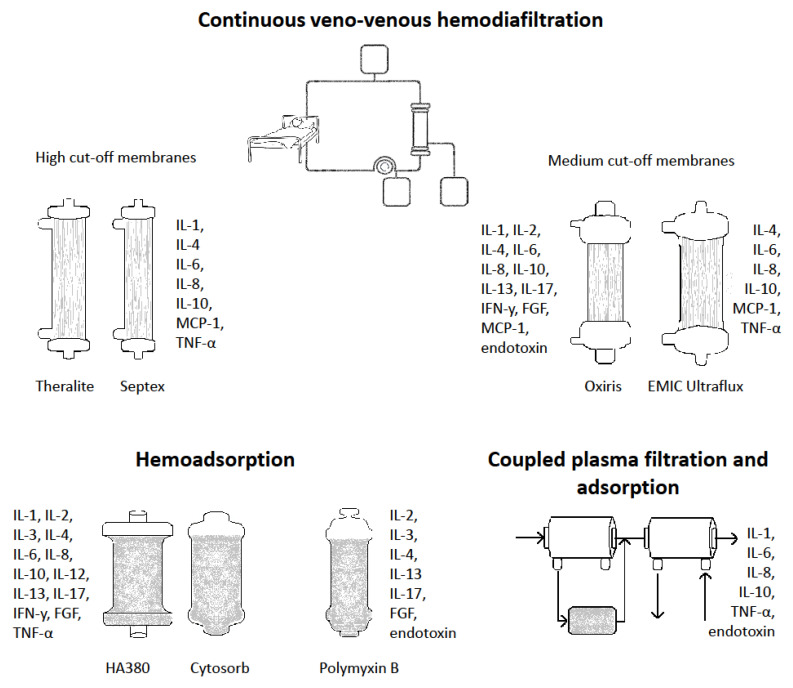
Current evidence on the clearance and adsorption of cytokines and endotoxins by different blood purification techniques. IL, interleukin; IFN, interferon; FGF, fibroblast growth factor; TNF, tumor necrosis factor; MCP, macrophage chemoattractive protein.

The general treatment strategy for a septic-shock-induced cytokine storm involves supportive care to maintain critical organ function and the elimination of exogenous and endogenous triggers for the abnormal activation of the immune system. Targeted immunomodulation (e.g., with the IL-6 antagonist, tocilizumab, in the management of COVID-19 infection) and nonspecific immunosuppression (e.g., corticosteroids) can be used to limit the collateral damage caused by the adversely activated immune system in conditions where the elimination of the triggering pathogen is not possible [23]. The removal of cytokines with blood purification therapies has been suggested as one method to improve immune homeostasis in sepsis, but there are limited outcome data regarding these approaches. However, the use of extracorporeal adsorption of inflammatory mediators using specifically developed filters, adsorption membranes, hemoperfusion sorbent cartridges or columns has lately attracted attention and these techniques are discussed in detail below (Figure 2) [24,25].

## 3. Acute Kidney Injury in Sepsis and Septic Shock

Acute kidney injury (AKI) is common in critically ill patients with or without sepsis. According to the 2012 Kidney Disease: Improving Global Outcomes (KDIGO) [26] guidelines, AKI is defined as an increase in serum creatinine by ≥0.3 mg/dl or ≥26.5 µmol/L in 48 h or ≥1.5 times baseline within the prior 7 days or a decrease in urine volume <0.5 mL/kg/h for 6 h [26]. AKI is further staged into three separate categories according to the severity of the kidney injury, with each increasing stage of AKI being associated with an increased risk of death and a greater need for renal replacement therapy (RRT).

Observational studies have demonstrated an AKI incidence of about 40% in patients with sepsis; the incidence has been as high as 41–64% in patients with septic shock [27,28,29]. Conversely, septic AKI has been estimated to account for almost 50% of all AKI patients treated in the ICU [30]. Risk factors associated with septic AKI include septic shock, high markers of disease severity, comorbidities, and blood-culture-positive infection [27,30,31]. RRT is commonly required in septic AKI patients, as every fifth patient required RRT in a substudy of a large international ICU audit [32]. Furthermore, patients with septic AKI were more likely to need RRT compared to those with non-septic AKI in that study. According to the KDIGO guidelines for the management of AKI, indications for the initiation of RRT in patients with AKI include severe and refractory electrolyte imbalance (e.g., marked hyperkalemia), acid–base disturbances (e.g., severe metabolic acidosis), or fluid dysbalance (e.g., high volume overload and/or low urine output), irrespective of the presence of sepsis [26]. The KDIGO guidelines further recommend favoring continuous RRT (CRRT), typically continuous veno-venous hemodialysis (CVVHD) or hemodiafiltration (CVVHDF), instead of intermittent hemodialysis (IHD) in patients with severely compromised hemodynamics. CRRT is better tolerated than IHD due to the slower and more gentle fluid removal [26]. Accordingly, CRRT has been the treatment of choice in hemodynamically unstable AKI patients with or without sepsis in large surveys on ICU clinical practice [33].

Concomitant AKI during sepsis is associated with worse patient outcomes than sepsis without AKI; similar observations have been made in patients with septic shock. The overall mortality associated with septic AKI has reached 30–70% depending on the study cohort [27,29,30]. Furthermore, AKI in patients with sepsis has been associated with prolonged ICU and hospital stays in survivors [27,30]. Risk factors for mortality in patients with septic AKI, as with sepsis alone, have included older age, comorbidities, higher disease severity and decreased urine output [34,35]. Nonetheless, a recovery of renal function has been observed in up to 40% of patients that survive septic AKI. In previous studies, the intensity of fluid resuscitation, prior kidney function, acute disease severity, and RRT requirement have been associated with renal recovery [36,37].

The pathophysiology of septic AKI is complex, multifaceted, and currently incompletely understood. Pro- and anti-inflammatory cytokine responses to severe infection contribute substantially to the development of septic AKI. The detrimental immune response to sepsis evokes adverse changes in both the systemic and renal circulations. In contrast to non-septic AKI, due to attenuated renal circulation during hypovolemic or cardiogenic shock, the outcomes in studies examining the renal circulation of experimental animals subjected to septic AKI have been more inconsistent and shown that the circulation can decrease, remain preserved, or increase [38]. Unfortunately, there is not much data available on renal circulation in human subjects with septic AKI. Recent observations from studies with small samples and that apply various methodologies, including phase contrast magnetic resonance imaging and Doppler ultrasound, have detected a decrease in renal blood flow in patients with septic AKI [39,40]. Moreover, renal microcirculatory alterations in the absence of macrovascular perfusion attenuation have been observed in an experimental rat model of endotoxemia. This suggests that adverse micro- and macrocirculatory changes are likely to be present during septic AKI [41]. Histopathological studies on the kidneys of patients dying due to sepsis and associated AKI have yielded nonspecific findings common to many severe illnesses associated with non-septic AKI [42]. 

## 4. Renal Replacement Techniques in Sepsis and Septic Shock

Some previous studies have suggested that RRT is associated with improved survival in sepsis and septic shock, but the current data remain inconsistent and controversial. Furthermore, the optimal RRT modality of choice, namely CRRT or IHD, and the timing of RRT initiation remain under debate in cases of septic AKI. There is no convincing evidence that increasing the effective dose of dialysis to over 25 mL/kg/h in CRRT confers any benefit in terms of mortality, duration of hospitalization, or renal recovery.

A recent meta-analysis conducted by Nash and coworkers assessed RRT modality in critically ill AKI patients with twenty-one eligible studies included in the analyses. No significant differences were observed in short-term mortality or kidney survival between CRRT, IHD, and sustained low-efficiency dialysis [43]. The authors, however, suggested that well-designed, adequately powered trials are needed to clarify the role of RRT modalities in the treatment of critically ill patients with AKI. In a retrospective multicenter trial, 25,750 critically ill AKI patients alive at hospital discharge were grouped according to initial RRT modality (CRRT or IHD). The study implied that renal recovery may be lower in patients treated with IHD as the primary modality for RRT [44]. The studies were, however, not restricted to patients with septic AKI.

In the Randomized Evaluation of Normal versus Augmented Level (RENAL) replacement therapy study, a total of 1508 critically ill AKI patients were randomized to receive CVVHDF with an effluent flow of either 40 mL/kg/h (higher-intensity therapy) or 25 mL/kg/h (lower-intensity therapy). No differences were observed between the treatment intensity groups in 90-day mortality or restoration of kidney function [45]. The IVOIRE study compared hemofiltration doses of 35 mL/kg/h and 70 mL/kg/h in 140 critically ill patients with septic shock but found no differences between the doses in terms of mortality, hemodynamic profile or organ function [46]. A randomized prospective study conducted by Park et al. examined the effect of dialysis dose on mortality in 212 critically ill patients with septic AKI by comparing conventional (40 mL/kg/h) and high (80 mL/kg/h) doses of CVVHDF. No differences in mortality were observed between the groups, but high doses of CVVHDF significantly reduced the concentrations of cytokines such as IL-6, IL-8, IL-1b, and IL-10, whereas this did not occur with the conventional dialysis dose [47]. A Cochrane review published in 2016 included six randomized controlled studies enrolling 3185 critically ill participants with AKI. The authors concluded that there was no significant difference between intensive versus less intensive CRRT on the mortality risk, the number of patients who were free of RRT after CRRT discontinuation, or in the length of hospital stay [48].

During recent years, several randomized controlled trials have examined the optimal timing of RRT initiation in critically ill AKI patients. The Effect of Early versus Late Initiation of RRT on Mortality in Critically Ill Patients with Acute Kidney Injury (ELAIN) trial and the Artificial Kidney Initiation in Kidney Injury (AKIKI) trial were published in 2016, but with opposing results [49,50]. The studies, however, differed in terms of study design, patient population and definitions of early and late initiation of RRT. The ELAIN study, a single-center trial with 231 mostly surgical patients (>94%), reported a significant mortality benefit in patients who were started on RRT within 8 h after reaching KDIGO stage 2 AKI (early group) compared to patients that were started on RRT at a later stage (within 12 h after reaching KDIGO stage 3 AKI, late group). The AKIKI trial was a multicenter trial with 620 mostly medical critically ill AKI patients, and found no differences in mortality between the early and the late groups. In the early group, patients were started on RRT within 6 h after reaching KDIGO stage 3 AKI, whereas in the late group RRT was only initiated when one or more of the classic criteria for dialysis initiation were met (severe hyperkalemia, acidosis or fluid retention) [49,50]. The Initiation of Dialysis Early Versus Delayed in the Intensive Care Unit (IDEAL-ICU) trial published in 2018 was a randomized multicenter trial that compared an early strategy with a delayed strategy for RRT initiation in severe AKI in patients in the initial phase of septic shock [51]. In the early strategy group, RRT was initiated within 12 h after documentation of failure-stage AKI (according to the risk, injury, failure, loss, and end-stage kidney disease (RIFLE) classification criteria). Patients assigned to the delayed-strategy group were started on RRT when at least one of the emergency criteria was met (hyperkalemia, metabolic acidosis, or fluid overload, including pulmonary edema). The trial was stopped prematurely due to futility after the second planned interim analysis. Among the 477 patients included in the analyses, 58% in the early strategy group and 54% in the delayed-strategy group had died at the 90 days follow-up (*p* = 0.38) [51]. The Standard versus Accelerated Initiation of Renal-Replacement Therapy in Acute Kidney Injury (STARRT-AKI) trial included 2927 critically ill AKI patients in the modified intention-to-treat analysis (1465 in the accelerated strategy group and 1462 in the standard strategy group) [52]. In the accelerated strategy group, clinicians were requested to start RRT within 12 h after their patients had fulfilled the KDIGO stage 2 AKI criteria. In the standard strategy group, clinicians were discouraged from initiating RRT until the development of hyperkalemia, metabolic acidosis or evidence of severe respiratory failure resulting from volume overload, or persistent unresolved AKI for at least 72 h after randomization. At 90 days, 43.9% of the patients in the accelerated strategy group and 43.7% of the patients in the standard strategy group had died (relative risk, 1.00; 95% confidence interval (CI), 0.93 to 1.09; *p* = 0.92). Among survivors at 90 days, 85 of 814 patients (10.4%) in the accelerated strategy group and 49 of 815 patients (6.0%) in the standard strategy group (relative risk, 1.74; 95% CI, 1.24 to 2.43) were dependent on maintenance dialysis, suggesting that an early initiation of RRT might have resulted in an attenuated renal prognosis in the initial survivors [52].

## 5. High Cut-Off Membranes

The cut-off of a membrane describes the smallest molecular weight of a solute that it retains. The cut-off value of high cut-off (HCO) membranes is generally considered to approximate to the molecular weight of albumin (65 kDa) and standard RRT low-flux membranes have cut-off values ranging between 10 and 30 kDa [53]. The medium cut-off membranes fall between the low cut-off and high cut-off membranes (35–45 kDa). Most cytokines have molecular weights between 8 and 60 kDa. The currently available HCO membranes include Septex (CRRT) and Theralite (IHD), marketed by Baxter. The available studies using HCO membranes in septic patients have not shown unequivocal improvements in patient outcomes. 

In an early small-scale study, Morgera et al. examined the effects of intermittent high-permeability hemofiltration (HPHF) on protein and coagulation status, hemodynamics, and the clearance of IL-6 and TNF-α in 16 patients with multiple organ failure induced by septic shock [54]. Intermittent hemofiltration was performed for 12 h every other day for 5 days using a polyamide 60 kDa cut-off hemofilter. A standard hemofilter with a larger surface area and a 30 kDa cut-off was used on the remaining days. They showed that although HPHF was hemodynamically well-tolerated and did not significantly affect the coagulation status, transmembrane protein loss was significant, at 7.6 g per 12 h treatment. The sieving coefficient for IL-6 was high (up to 0.90) throughout the study and although HPHF seemed to significantly lower IL-6, the filtration capacity for TNF-α was poor. In another study, the same research group compared the effects of different treatment modalities and dialysis doses for CRRT with HCO membranes (P2SH; effective surface area 1.1 m^2^, steam sterilized; Gambro Corporate Research, Hechingen, Germany) on clinical parameters, protein loss, and circulating cytokine levels [55]. In the study, twenty-four patients with septic AKI were randomly allocated to either continuous veno-venous hemofiltration (CVVHF) with an effluent rate of 1000 mL/h or 2500 mL/h or CVVHD with a dialysate flow rate of 1000 mL/h vs. 2500 mL/h (four groups). In the whole study population, APACHE II scores improved during RRT and organ dysfunction declined, with no differences between the treatment groups. A significant reduction in plasma levels of interleukin-1 receptor antagonist (IL-1ra) and IL-6 was observed in those with high baseline concentrations of IL-1ra and IL-6. The clearance of IL-1ra was higher with CVVHF compared to CVVHD, whereas no differences in clearances were observed for IL-6. Increasing effluent or dialysate flow significantly increased cytokine clearances. In a further randomized pilot study, Morgera et al. compared the use of the PS2H HCO membrane for hemofiltration with conventional hemofiltration (filter cut-off 30 kDa) in 30 patients (20:10). The norepinephrine requirement was shown to be reduced over time and the clearance rates for IL-6 and IL-1ra were significantly higher in the HCO group [56].

There is evidence that HCO membranes can efficiently lower cytokine levels in patients with high pre-treatment plasma values, whereas in those with low baseline levels, the plasma cytokine levels may even rise during HCO RRT [55]. In a retrospective case-control study of 24 patients with septic shock, published in 2016, Chelazzi and coworkers compared the use of CVVHD with an HCO membrane to CVVHDF with a medium cut-off membrane (AN-69-ST, Gambro Lundia AB, Lund, Sweden). The authors reported a shorter requirement for vasopressors and mechanical ventilation, a shorter duration of ICU stay, and a lower hospital mortality in those patients receiving HCO CVVHD [57]. The later published randomized studies, however, have not shown any mortality benefit with HCO RRT in septic shock. In an Australian single-center randomized double-blind study comparing HCO and standard CVVHF in 76 (36 vs. 36) patients with vasopressor-dependent AKI, no differences between the groups were observed in terms of the duration of vasopressor requirement or hospital and ICU mortality [58]. In an analysis of cytokine clearance in the same study cohort, the sieving coefficient values and clearances were higher for IL-6 and IL-8 with an HCO membrane, as was combined cytokine mass removal. However, there were no significant differences in the reduction of cytokine plasma levels between the HCO and standard groups [59].

The randomized High Cut-Off Sepsis (HICOSS) trial aimed to examine the benefits of an HCO membrane for CVVHD in 120 patients with septic shock and concomitant AKI. The study was, however, terminated early after the first interim analysis, including only 81 patients, detected no difference in 28-day mortality between the HCO and standard groups. Furthermore, no differences were observed in the duration of ICU stay or mechanical ventilation or the need for vasopressors [60]. 

## 6. Medium Cut-Off Membranes

### 6.1. Ultraflux EMIC2

The Ultraflux EMIC2 filter (Fresenius Medical Care, Bad Homburg, Germany) for CRRT is a medium cut-off polysulfone-based membrane with a cut-off of 45 kDa and a sieving coefficient of 0.9 for β2-microglobulin (17 kDa) and 0.01 for albumin. Recent studies have examined the performance of the EMIC2 hemofilter for the clearance and reduction in plasma cytokines, including TNF-α, ILs 1α, 1β, 2, 4, 6, 8, and 10, vascular endothelial growth factor (VEGF), macrophage chemoattractive protein 1 (MCP-1), and endothelial growth factor (EGF), in comparison with a standard low cut-off hemofilter in patients with septic AKI [61,62,63].

Eichhorn and coworkers performed a combined in vitro and clinical study to examine the effects of CVVHD with the EMIC2 or a standard low cut-off hemofilter (AV-1000, Fresenius Medical Care, Bad Homburg, Germany) [61]. The in vitro clearance values of IL-6 (26 kDa) and IL-10 (17 kDa) were significantly higher for the EMIC2 filter. However, no differences were observed in the clearance of the low-molecular-weight cytokine IL-8 (8 kDa). The in vivo part of the study examined 30 patients with septic AKI randomized to CVVHD with EMIC2 (14 patients) or AV-1000 (16 patients). The clearance rates of IL-6 and IL-10 were higher for EMIC2 but the mean plasma cytokine concentrations decreased similarly between the treatment groups. Balgobin et al. compared the use of EMIC2 and AV-1000 in a randomized cross-over setting. They showed that plasma levels of neither the proinflammatory cytokines TNF-α, IL-1α, IL-1β, IL2, IL-6, and IL-8, nor the anti-inflammatory cytokines IL-4 or IL-10, were lowered during 24 h of CRRT, irrespective of which hemofilter was employed [62]. In line with these earlier studies, Lumlertgul and coworkers recently detected a reduction in plasma levels of multiple cytokines during the first 48 h of CVVHD with EMIC2. However, the CVVHD clearance of the tested 12 cytokines in proportion to plasma levels was low. They concluded that endogenous cytokine metabolism may be a more important determinant of plasma levels than the CRRT-associated clearance [63].

### 6.2. Oxiris

The oXiris^®^ haemofilter (Baxter, Deerfield, IL, USA) is a medium cut-off (35–40 kDa) polyacrylonitrile methalylsulfonate (AN69)-based membrane, coated with positively charged polyethyleneimine (PEI) and pre-grafted with 4500 IU of heparin per m^2^. The positively charged PEI coating enables the adsorption of negatively charged endotoxins in addition to the cytokine adsorption and clearance in addition to renal replacement properties attributable to the AN69 hemofilter membrane. 

Malard et al. examined the effects of in vitro hemoperfusion with the oXiris^®^ hemofilter, CytoSorb^®^, and Toraymyxin^®^ (polymyxin B) hemoperfusion devices. Heparinized human plasma was pre-incubated with pathological quantities of cytokines and filtered in a closed-loop circulation model for 2 h and the removal of 27 cytokines for each device was examined. Endotoxin removal by oXiris and Toraymyxin was assessed using a six-hour hemoperfusion. Endotoxin (lipopolysaccharide) removal was most rapid with Toraymyxin, whereas it was negligible with CytoSorb. At the end of hemoperfusion, there was no significant difference between the endotoxin removal rates using oXiris and Toraymyxin. The removal rates of cytokines and other inflammatory mediators were similar between Oxiris and CytoSorb [64]. Broman et al. compared the endotoxin and cytokine reducing properties of Oxiris and a standard medium cut-off hemofilter (M150ST, Baxter, USA) for CRRT in a randomized cross-over double-blind study including 16 patients with septic shock. They reported that endotoxin levels decreased with Oxiris, whereas they remained stable with the standard hemofilter. Levels of TNF-α, IL-6, IL-8, and interferon y, decreased more with the Oxiris filter [65]. 

Currently, data on the clinical effects of the Oxiris hemofilter in sepsis and septic shock are mainly based on anecdotal findings of case series and retrospective cohorts. Randomized controlled studies showing improved prognosis are lacking. Shum and coworkers examined the development of SOFA scores during CVVHF with the Oxiris filter compared to a matched historical cohort treated with standard CVVHF. They reported a reduction of 37% in SOFA scores at 48 h post-initiation of Oxiris–CVVHF versus an increment of 3% in the historical controls but no differences were observed in mortality between the two cohorts [66]. In a retrospective cohort of 60 patients with sepsis or septic shock and CVVHD using the Oxiris membrane, there were improvements in hemodynamics and vasoactive requirements and SOFA scores became reduced during the first 48 h of CVVHD. At the same time, reductions were observed in the levels of IL-6, IL-10, procalcitonin, and endotoxin activity. The study did not report mortality in the cohort and it is not clear whether some patients had died before the end of the 48 h of CRRT [67]. In another retrospective cohort from two French centers, 31 patients with septic shock and AKI received CRRT with the Oxiris membrane between 2014 and 2019. The researchers observed a lower-than-expected hospital mortality for patients with the highest severity of illness according to the Simplified Acute Physiology (SAPS) scores but did not detect a reduction in SOFA scores during the first 48 h of CRRT with Oxiris [68].

## 7. Hemoadsorption

### 7.1. Polymyxin B Hemoadsorption

Endotoxin (lipopolysaccharide) is an essential component of Gram-negative bacteria cell walls. Hemoadsoption using polystyrene fibers with immobilized Polymyxin B which possesses a high affinity for endotoxins was first approved in Japan in 1994 and later awarded the CE designation in 1998. Polymyxin B is a polycationic antibiotic which binds the lipid A portion of the endotoxin and neutralizes its toxicity [69]. The efficacy of polymyxin B hemoperfusion (PMH) on patient survival in septic shock has been studied in several randomized trials, but a survival benefit remains to be conclusively demonstrated.

The Early Use of PMH in Abdominal Septic Shock (EUPHAS) trial was the first randomized controlled trial examining the effects of PMH on 28-day mortality. All study patients had received emergency surgery for intra-abdominal sepsis or septic shock due to Gram-negative bacteria [70]. Mean arterial pressure increased and SOFA scores declined with PMH but not with conventional treatment, and mortality was lower in the PMH group (32% vs. 53%, adjusted HR 0.36, 95% CI 0.16–0.80). The study was stopped early due to the statistically significant survival benefit associated with PMH and the number of enrolled patients was therefore limited to only 64. A French multicenter, randomized controlled trial performed in 18 ICUs enrolled 243 patients with septic shock within 12 h after emergency surgery for peritonitis related to intestinal perforation. The PMH group received conventional therapy plus two PMH treatments. Mortality at 28 and 90 days was similar in the PMH and the standard therapy groups, contradicting the results of the EUPHAS trial [71]. The North American Effect of Targeted Polymyxin B Hemoperfusion on 28-day mortality in Patients with Septic Shock and Elevated Endotoxin Level (EUPHRATES) randomized multicenter trial examined 450 patients with septic shock and high endotoxin activity levels. Survival was compared in patients treated with two 2 h PMH sessions within 24 h and those receiving conventional care; no differences were observed in 28-day mortality between the study groups [72]. A post-hoc analysis of the EUPHRATES data revealed a mortality benefit when patients with very high endotoxin activity levels (≥0.90) were excluded from the analyses (26.1% vs. 36.8%, *p* = 0.047). The researchers suspected that in the case of extremely high endotoxin levels, two 2 h PMH sessions were insufficient to achieve any significant endotoxin clearance and thus a survival benefit [73].

In a recent meta-analysis including 13 trials and 1163 patients, PMH was associated with a lower mortality at the longest follow-up available compared to standard treatment. However, the analysis was limited by its very high heterogeneity. No differences were observed in 30-day mortality. The low-risk of bias studies (three trials, 745 patients) observed no differences in mortality [74]. The ongoing Safety and Efficacy of Polymyxin B Hemoperfusion for Endotoxemic Septic Shock in a Randomized, Open-Label Study (TIGRIS) (ClinicalTrials.gov identifier: NCT03901807) is currently enrolling patients (target of 150 patients) with septic shock. The inclusion criteria include endotoxin activity levels of 0.60–0.89 to corroborate the results of the EUPHRATES trial post-hoc analysis on the survival benefit of PMH.

### 7.2. CytoSorb

CytoSorb^®^ (CytoSorbents, Monmouth Junction, NJ, USA) is an extracorporeal blood purification device targeting the removal of cytokines in the low- to middle-molecular-weight range (10–55 kDa) using highly porous pyrrolidone-coated polystyrene-divinyl-benzene polymer beads. CytoSorb was awarded the CE marking in Europe in 2011 for the management of conditions associated with elevated cytokinemia, such as sepsis and septic shock. The high (>95%) removal rate of pro- and anti-inflammatory cytokines including IL-6, IL-10, TNF-α, and IFN-γ is based on surface adsorption, pore capture, and the large surface area of the CytoSorb cartridge. CytoSorb has been superior in efficacy when compared to the high cut-off filter EMiC2 and endotoxin removal cartridge polymyxin B but similar to that of the Oxiris cytokine filter [64,75]. When compared to the Oxiris filter and the Toraymyxin (polymyxin B) device, however, CytoSorb did not remove endotoxins and has been associated with mild plasma protein loss and thrombocytopenia [64,76]. CytoSorb has been demonstrated to be safe and to decrease vasopressor requirements and plasma cytokine levels or lactatemia in patients with sepsis or septic shock and normal kidney function as a stand-alone hemoadsorption therapy. Similar results have been demonstrated with CytoSorb as an adjunctive blood purification therapy in septic patients receiving dialysis and/or extracorporeal membrane oxygenation (ECMO) [77,78,79]. However, one prospective study examining 45 patients with sepsis or septic shock and one large retrospective study on 100 patients with septic shock failed to demonstrate a reduction in vasopressor dosing requirements, although there was a decrease in serum lactate levels after CytoSorb therapy. Both studies detected a decrease in SOFA and APACHE-II scores after CytoSorb therapy in the survivor groups and observed that mortality was lower than predicted by the APACHE-II score [80,81]. In another study by Schittek et al., 43 patients with septic shock and CRRT-treated AKI were prospectively enrolled to receive CytoSorb adjunction therapy and compared with a historical cohort of 33 patients. No difference was observed in survival between the groups [82].

Patient outcomes were also assessed by Brouwer et al. in two consecutive studies on a propensity-score-weighted retrospective cohort of 116 patients with septic shock and CRRT-dependent AKI. A total of 67 patients received CytoSorb therapy with the other 49 patients receiving CRRT and standard care. At baseline, the CytoSorb group had higher serum lactate levels and vasopressor dosing compared to the control group and the groups were weighted with stabilized inverse probability of treatment weights (sIPTW). In the sIPTW analysis, the patients who received CytoSorb had better 28-day and one-year survival compared to the CRRT group in the first and second study, respectively [83,84]. Similarly, Rugg et al. demonstrated a survival benefit associated with CytoSorb therapy in another retrospective cohort study exploring propensity-score-matched patients (42 receiving CytoSorb vs. 42 controls) with septic shock and CRRT-treated septic AKI [85]. The largest study on septic shock patients and CytoSorb hemoadsorption to date was published in 2021 by Kogelmann et al. These authors evaluated the accuracy of a new dynamic scoring system in predicting mortality in this population as well as guiding the timing of CytoSorb therapy initiation. Altogether, 502 patients were recruited in this retrospective study, with 198 receiving CytoSorb along with CRRT. Out of the 304 patients receiving standard therapy, only 69 patients required CRRT. Although a direct comparison between CytoSorb-treated patients and controls was not reported, improved survival was observed in the CytoSorb group in patients who initiated CytoSorb hemoadsorption earlier (≤12 h vs. >24 h, *p* = 0.037). However, CytoSorb therapy was not associated with 56-day survival (*p* = 0.095) in a multivariable analysis of a subset of patients who received CytoSorb or CRRT and standard care [86]. Finally, CytoSorb therapy was not associated with improved survival in a recent meta-analysis assessing the effect of different extracorporeal blood purification techniques on mortality in patients with sepsis [87]. A clinical trial assessing the efficacy of CytoSorb hemoadsorption compared to standard medical therapy in terms of shock reversal in patients with early refractory septic shock is ongoing (ClinicalTrials.gov identifier: NCT04742764).

### 7.3. HA380

HA380 (Jafron Biomedical Co., Ltd., Zhuhai, China) is a CE-labeled hemoadsorption cartridge developed to treat patients with septic shock. It contains hemocompatible, porous polymeric beads that adsorb cytokines and mid-molecular-weight toxins onto the surface of the beads. The range of molecule sizes adsorbed by the HA380 lies in a range between 10–60 kDa [88]. It has been reported that HA380 can adsorb several cytokines including IL-1, IL-6, IL-8, IL-10, and TNF-α [89] in addition to complements, free hemoglobin, and other molecules over a wide range of molecular weight [88]. A clinical trial examining the use of the HA380 hemoadsorption in tandem with CVVHDF using the Oxiris membrane compared to mere Oxiris CVVHDF (ClinicalTrials.gov identifier: NCT04997421) and a large observational study examining the effects of HA380 on cytokine levels (ClinicalTrials.gov identifier: NCT04306419) are ongoing.

## 8. Plasma Exchange in the Treatment of Septic AKI

### 8.1. Plasma Exchange

Plasmapheresis or plasma exchange (PE) is an extracorporeal therapy in which the patient’s plasma is removed and exchanged for fresh frozen plasma and/or albumin and crystalloid solution. During the course of PE, potentially injurious autoantibodies and cytokines are removed and the patient receives regular donor plasma without the excess molecules, thus ameliorating the targeted inflammatory condition. The application of PE in patients with sepsis has been studied previously with the hypothesis that the removal of detrimental cytokines should reduce the adverse immune response to infection. However, the results on patient outcomes have been inconclusive and research focus in blood purification techniques in septic patients has shifted more towards newer technologies, such as special dialysis filters and hemoadsorption devices. 

Prior studies have shown that PE is relatively safe and can restore hemodynamic stability, fluid balance and organ dysfunction in patients with septic shock, but no evidence for improved patient survival has emerged [90,91]. Only one randomized controlled trial on the efficacy of PE in adult patients with sepsis or septic shock has been performed by Busund et al., demonstrating a lower 28-day all-cause mortality in the PE arm [92]. However, a meta-analysis assessing the effect of PE on survival in patients with sepsis or septic shock detected an unclear-to-high risk of bias, and criticized the low sample sizes in these studies (including the study of Busund et al.). Furthermore, it was reported that PE was not associated with any survival benefit [93]. Recently, a case report on the use of a new technique, i.e., continuous PE in combination with dialysis in the management of sepsis, was published; this may represent a new approach for investigating the feasibility of PE in the treatment of sepsis [94]. Nonetheless, the latest update of the Surviving Sepsis Campaign: International Guidelines for Management of Sepsis and Septic Shock: 2016 does not recommend the use of PE in the management of sepsis due to insufficient evidence [95].

### 8.2. Coupled Plasma Filtration and Adsorption

Coupled plasma filtration and adsorption (CPFA) is a two-stage blood purification technique that was developed for the management of sepsis in the late 1990s. The CPFA circuit consists of a plasma filter and a CRRT dialysis hemofilter connected in series. The plasma filter diverts the plasma of the patient through a hydrophobic styrenic polymer resin adsorbent cartridge with a high affinity for inflammatory mediators (such as IL-1β, TNF-α, IL-6, IL-8, C3a desArg, and IL-10) and endotoxins. Then, the filtered plasma is returned to the patient through a dialysis hemofilter for further solute and fluid removal [96]. Circuit anticoagulation can be performed with either regular heparin-based anticoagulants or regional citrate anticoagulation, as septic patients with AKI are at an increased risk of bleeding similarly to patients with concomitant traumatic or burn injuries [97,98]. 

In a pilot study conducted by Ronco et al. in 2002, CPFA was demonstrated to improve hemodynamics, decrease vasopressor requirement and TNF-α levels in a small sample of patients with septic shock as compared to CVVHDF [99]. Formica et al. reported similar findings in a similarly sized prospective study on septic shock patients with or without septic AKI [100]. Furthermore, favorable effects on patient hemodynamics and inflammatory mediator levels were observed in a more recent prospective study comparing CFPA with high-volume hemofiltration in fourteen patients with septic shock and multiple organ dysfunction syndrome [101]. However, in an earlier experimental study on pigs with peritonitis-induced septic shock treated with CPFA, no improvement in hemodynamic or biochemical measures was observed as compared to supportive therapy [102].

There is very little clinical outcome data about the pros or cons of CPFA in patients with sepsis. The COMPACT trial randomized 192 patients with septic shock to receive CPFA vs. no CPFA with standard therapy. No benefit was observed in terms of mortality or other clinical outcomes. However, a subgroup analysis showed a survival benefit in patients treated with a higher CPFA dose [103]. A subsequent study designated as COMPACT 2 which was intended to explore the effects of higher-dose CPFA in patients with septic shock had to be prematurely terminated due to increased mortality in the CPFA arm observed in the predetermined interim analysis. The subsequent events led to the early discontinuation of another similarly designed randomized study, the ROMPA trial, before target sample size was achieved leading to inconclusive results [104]. Thus, a meta-analysis on the effects of CPFA on patients with severe sepsis including 17 eligible studies concluded that further evidence is required before one can draw definite conclusions. However, fewer deaths were observed in patients who received CPFA compared to other treatments [105].

More recently, CPFA was explored in severe burn patients with septic shock and AKI in a retrospective cohort study. The study demonstrated a survival benefit compared to RRT alone, although more data in this set-up will be needed [106]. Furthermore, the concept of combining CPFA with another blood purification technique was adopted in a small cohort of postoperative patients with severe sepsis. The patients in the intervention arm received CPFA in combination with polymyxin B hemoadsorption in a single circuit. The researchers observed an amelioration in hemodynamics and a decrease in inflammatory marker activity without any severe adverse effects in patients who received CPFA in combination with PMH [107]. 

## 9. Conclusions

Sepsis and septic shock remain common conditions and, especially with concomitant septic AKI, are associated with substantial morbidity and mortality despite modern critical care and the recently introduced blood purification techniques. Several advanced RRT filters and hemoadsorption devices have been demonstrated to provide effective clearance of proinflammatory cytokines and/or endotoxins in patients with sepsis or septic shock. However, definitive data that these blood purification techniques confer any survival benefit remain elusive. Thus, the latest Surviving Sepsis Campaign: International Guidelines for Management of Sepsis and Septic Shock issued in 2016 do not provide any recommendations on the use of blood purification techniques in patients with sepsis. Currently the indications for RRT initiation in septic AKI do not differ from AKI caused by other etiologies. The use of special adsorptive techniques may be considered for adjunctive therapy on a case by case basis. The lack of convincing outcome data is actually not surprising, as in recent years several randomized controlled trials evaluating a variety of interventions in critically ill patients with or without sepsis have failed to detect any survival benefit. This highlights the difficulty in assessing the effects of interventions in large cohorts of patients with different backgrounds, i.e., the interventions may be beneficial in some and hazardous in others, leading to an inconclusive overall effect. Nevertheless, several of the ongoing trials on different blood purification filters and hemoadsorption devices are likely to shed further light on optimal patient and technique selection to achieve better outcomes in the management of sepsis and septic shock. 

## Data Availability

Not applicable.
